# Individual Differences in Boldness Influence Working Memory and Stress‐Induced Repetitive Behaviors in Zebrafish

**DOI:** 10.1111/ejn.70173

**Published:** 2025-07-02

**Authors:** Barbara D. Fontana, Hevelyn S. Moraes, Camilla W. Pretzel, Khadija A. Mohammed, Julia Canzian, Carla D. Bonan, Matthew O. Parker, Denis B. Rosemberg

**Affiliations:** ^1^ Laboratory of Experimental Neuropsychobiology, Department of Biochemistry and Molecular Biology Federal University of Santa Maria Santa Maria Rio Grande do Sul Brazil; ^2^ Graduate Program in Biological Sciences: Toxicological Biochemistry Federal University of Santa Maria Santa Maria Rio Grande do Sul Brazil; ^3^ Laboratório de Neuroquímica e Psicofarmacologia, Programa de Pós‐Graduação em Biologia Celular e Molecular, Escola de Ciências da Saúde e da Vida Pontifícia Universidade Católica do Rio Grande do Sul Porto Alegre Rio Grande do Sul Brazil; ^4^ Surrey Sleep Research Centre University of Surrey Guildford UK; ^5^ The International Zebrafish Neuroscience Research Consortium (ZNRC) Slidell Louisiana USA

**Keywords:** alarm substance, FMP Y‐maze, individual differences, novel tank diving test, stress reactivity

## Abstract

Individual differences in traits, such as boldness, significantly impact survival and adaptability by shaping responses to environmental challenges, including stress. Bolder individuals take greater risks to access resources and often display higher impulsivity, affecting behavioral domains like memory and cognition. Coping mechanisms in response to stressful situations may also differ between bold and shy individuals, with bold fish tending towards impulsive, less inhibited responses, whereas shy fish adopt cautious, controlled strategies. Despite these distinctions, the connections between boldness, stress reactivity, cognitive performance, and maladaptive behaviors remain poorly understood. Here, we used the zebrafish ( *Danio rerio* ) to examine these relationships. Boldness was assessed using the novel tank diving test, classifying fish as bold or shy based on overall and vertical exploratory activity. We then exposed fish to conspecific alarm substance (CAS), an acute naturalistic stressor, and assessed cognitive outcomes in the FMP Y‐maze. Our results revealed distinct responses, with bold zebrafish showing poorer working memory performance and higher levels of repetitive behaviors following stress compared to shy fish, underscoring the impact of individual differences on stress reactivity and cognition. A positive correlation between boldness and repetitive behaviors was found for both control and CAS‐exposed fish, indicating that boldness influences the escape strategies adopted by different phenotypes on the FMP Y‐maze, especially in patterns associated with repetitive behaviors. These findings advance our understanding of how boldness influences stress responses and cognitive outcomes, providing a framework to examine resilience, impulsivity, and repetitive behaviors in translational neurobehavioral research.

## Graphical abstract

This study investigates the influence of boldness on zebrafish cognition and repetitive behaviors, focusing on the role of stress. Boldness was measured using the novel tank diving test and quantified with a boldness index. Zebrafish were exposed to a naturalistic stressor, conspecific alarm substance (CAS), before behavioral assessment in the free movement pattern (FMP) Y‐maze. Bold individuals exhibited distinct behavioral responses to stress, including poorer working memory and increased repetitive behaviors, highlighting the complex relationship between boldness and stress‐induced cognitive and behavioral outcomes.

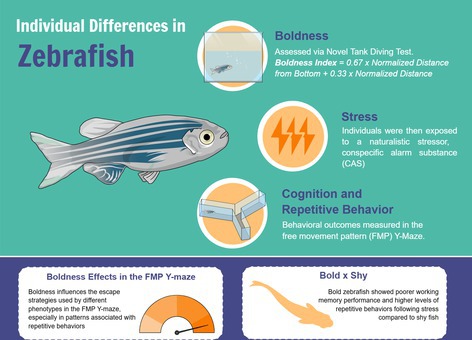

**Abbreviations**
ANOVA: analysis of variance
ASD: autism spectrum disorder
CAS: conspecific alarm substance
CPP: conditioned place preference
CTRL: control group
FMP Y‐maze: free movement pattern Y‐maze
NTT: novel tank diving test
RM‐ANOVA: repeated measures analysis of variance
SEM: standard error of the mean

## 1 Introduction

Individual differences are essential for species survival, influencing adaptability to changing environments and shaping responses to threats (Sansom et al. 2009; Shaw 2020). Boldness is a behavioral trait broadly defined as a consistent individual tendency to take risks and explore novel environments, directly influencing both survival and reproductive success (Smith and Blumstein 2008). Although bold individuals face an increased risk of predation, this propensity to take greater risks often affords them better access to food and other resources (Dammhahn and Almeling 2012; Hulthén et al. 2017; Ólafsdóttir and Magellan 2016). As well as playing an important role in survival, boldness is also relevant for psychiatric research, as it is linked to stress reactivity, anxiety, behavioral flexibility, and coping strategies (Atwell et al. 2012; Hamilton et al. 2021; Perkins et al. 2022). In humans, boldness can also affect cognition, decision‐making, and memory; bold individuals often show significant impairments on cognitively demanding tasks due to their high levels of impulsivity (Perales et al. 2009; Romer et al. 2009; Yancey et al. 2022). Therefore, understanding boldness can provide relevant insights on how individuals differ in response to stress and how this trait can affect other behavioral aspects, such as repetitive behaviors and cognition.

Repetitive behaviors, often defined as repeated and invariant actions occurring in the absence of clear goals, are also observed as a response to stress‐inducing environments (Langen et al. 2011; Zhang et al. 2022). These behaviors can be considered a maladaptive coping mechanism that in humans is often observed in psychiatric disorders, such as autism spectrum disorder (ASD) (Ohtsubo et al. 2024). Their presence can reflect reduced behavioral flexibility and impaired cognitive control, which are central features in various mental health conditions (D'Cruz et al. 2013; Mostert‐Kerckhoffs et al. 2015). Investigating repetitive behaviors in animal models thus provides a translational window into understanding how stress and individual traits interact to shape cognitive function and coping styles. Together, boldness and repetitive behaviors offer a powerful framework for modeling key behavioral and cognitive mechanisms underlying psychiatric conditions, such as anxiety disorders, ASD, and impulsivity‐related disorders.

Animal species that exhibit naturally occurring bold and shy behavioral traits, like the zebrafish ( *Danio rerio* ), are particularly useful for examining how individual differences shape stress reactivity, coping mechanisms, and cognitive processes. Bold and shy zebrafish display distinct behavioral responses to stressors, such as the conspecific alarm substance (CAS), a fear cue released during predator attacks. For example, bold zebrafish have lower responses to stress by displaying decreased anxiety‐like behaviors and lower cortisol levels in response to CAS compared to shy fish (Baker and Wong 2019), demonstrating that boldness affects animals' reactivity to a potential threat. CAS exposure is known for triggering stress‐related responses at both physiological and behavioral levels by increasing whole‐body cortisol levels and anxiety/fear‐related responses (Barkhymer et al. 2019; Borba et al. 2025; Li et al. 2024; Mezzomo et al. 2019), making it a valuable tool for studying stress reactivity in zebrafish. Thus, given the well‐characterized behavioral traits of zebrafish, this species is an excellent model for studying individual differences and can help researchers to better understand how boldness can shape stress responses in different behavioral domains.

Using well‐established behavioral paradigms in zebrafish, this study aimed to explore the relationship between boldness, stress reactivity, repetitive behaviors, and working memory. Boldness was first assessed through the novel tank diving test (NTT), a widely used behavioral assay in zebrafish research. Although the NTT has often been employed as a measure of anxiety‐like behavior (Egan et al. 2009; Levin et al. 2007), it has recently been validated as a method to explore boldness in zebrafish (Mustafa et al. 2019; Rajput et al. 2022; Thörnqvist et al. 2019). Boldness, in this context, is characterized by exploratory behavior, including time spent in the upper zone of the tank, reflecting increased exploration and risk‐taking tendencies. This allowed us to classify zebrafish as either bold or shy based on their overall exploratory activity. Based on prior findings that bold zebrafish exhibit distinct stress reactivity patterns, including lower cortisol levels and reduced behavioral inhibition in response to CAS, we hypothesized that bold individuals would exhibit altered patterns of repetitive behaviors and working memory performance under acute stress compared to shy individuals. Furthermore, the tendency of bold individuals to adopt impulsive strategies may shape their responses to stress and associated cognitive outcomes. Thus, we investigated how these individual traits influenced stress responses and performance in the free movement pattern (FMP) Y‐maze, a task designed to evaluate working memory and repetitive behaviors, using CAS as an acute stressor model. Our aim was to characterize how bold and shy zebrafish respond to acute stress, particularly regarding working memory and repetitive behaviors, to provide a deeper understanding of the interaction between behavioral traits, stress, and cognitive outcomes. Specifically, we hypothesized that bold individuals would show pronounced cognitive impairments and increased repetitive behaviors following acute stress exposure compared to shy individuals.

## 2 Material and Methods

### 2.1 Animal Husbandry

Zebrafish (*n* = 46; 4–6 months old; 24:22 female: male fish) were obtained from a local supplier (Hobby Aquários, RS, Brazil). The number of animals per group was calculated a priori (d = 0.5, power = 0.75, alpha = 0.05) considering extensive published work from our laboratory using zebrafish as a translational model and based on primary outcomes, which resulted in a sample size of 12 animals per group (Canzian et al. 2017; Cleal et al. 2021; Fontana et al. 2021b; Mezzomo et al. 2019). Animals used were from the short‐fin phenotype, kept under constant filtration and aeration in 40‐L tanks filled with non‐chlorinated water (2 fish/L). The water temperature was set at 27°C (±1°C), pH 7.0–7.2, dissolved oxygen at 6.0 ± 0.1 mg/L, and total ammonia at < 0.01 mg/L. Fish were acclimatized in laboratory conditions for at least 14 days before the experiment. Fish were fed three times a day with commercial flake fish food (Alcon BASIC, Alcon, Brazil). Fluorescent lamps provided adequate lighting on a 14/10 light/dark photoperiod (lights on at 7:00 a.m.). Behavioral tests were performed between 9:00 a.m. and 4:00 p.m., and the water was changed between each subject (Fontana et al. 2021a).

### 2.2 Experimental Design

**FIGURE 1:**
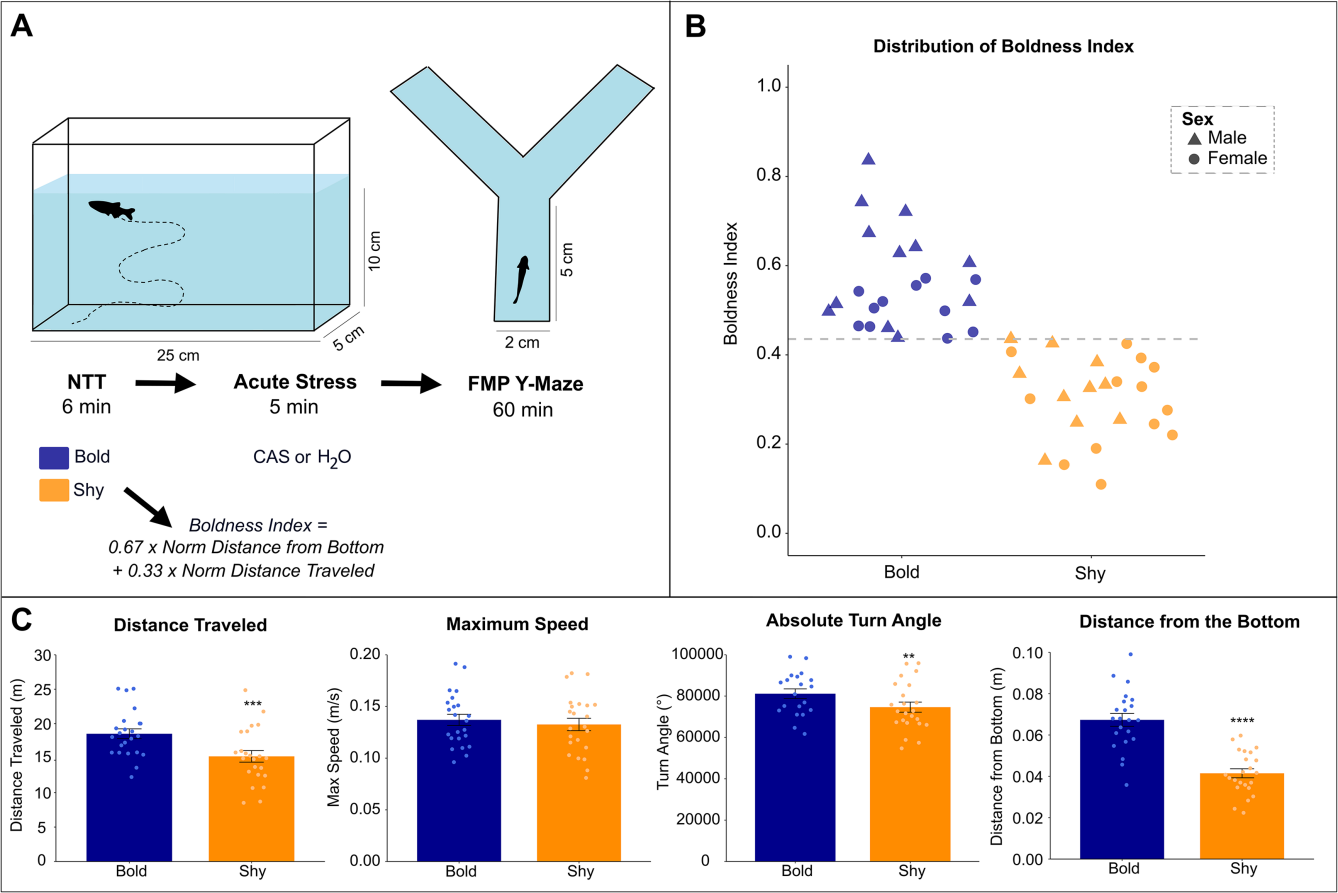
Experimental design and characterization of boldness with the respective main behaviors of bold and shy individuals. (A) Illustration of the experimental setup detailing the sequence of behavioral assessments, including the novel tank test (NTT) for boldness assessment, followed by acute stress exposure, and the free movement pattern (FMP) Y‐maze task to evaluate working memory, cognitive flexibility, and repetitive behaviors. Experimental groups (bold and shy) were treated with conspecific alarm substance (CAS) or water (H_2_O). The boldness index was calculated based on the normalized distance from the bottom and the total distance traveled. Shy and bold groups were defined based on boldness index after all behavioral recordings; thus, 24 animals were exposed to CAS, and 24 animals were exposed to water to mimic handling. (B) Distribution of boldness index scores across groups (bold and shy) with differentiation by sex (male and female). (C) Bar plots showing difference between bold and shy behavior in the NTT (total distance traveled, maximum speed, absolute turn angle, and average distance from the bottom). Data are presented as mean ± SEM analyzed by Student's *t*‐test. Asterisk represent significant difference compared to bold fish (**p < 0.01, ***p < 0.001, ****p < 0.0001; *n* = 23).

To evaluate their boldness phenotypes, individual zebrafish were first submitted to the NTT for 6 min. Zebrafish were randomly selected from two holding tanks and randomly assigned to either the control (CTRL) or CAS groups for each experimental replicate. Experiments were conducted across five experimental replicates, ensuring that each fish was tested only once. Animals were exposed to water (CTRL group) or conspecific alarm substance (CAS group), an acute stressor, for 5 min in 500‐mL beakers. Each fish was exposed to a unique CAS dilution prepared individually, and a fresh beaker was used for each animal. Beakers were thoroughly rinsed three times between fish to prevent cross‐contamination, including for the CTRL group. Then, to evaluate cognitive flexibility, working memory, and repetitive behaviors, animals were tested on the FMP Y‐maze for 60 min (Figure 1A). To control the potential effects of handling stress, all animals underwent identical procedures throughout the experimental timeline. Additionally, previous studies have shown that handling associated with multiple behavioral tests or brief transfers does not significantly alter zebrafish boldness, memory, and cognition (Fontana et al. 2022, 2021b). Experiments were performed in a randomized order of treatments and behavioral recording. Two animals were excluded due to a tracking error. Behaviors were recorded using a Logitech C920 Pro HD Webcam at 30 frames/s. Immediately after the FMP Y‐maze recordings, animals were anesthetized using the rapid cooling (4°C) method and euthanized by decapitation. Sex was initially determined by analyzing sexually dimorphic physical characteristics (color and body shape) and confirmed postmortem by gonadal dissection (Yossa et al. 2013). All behavioral recordings were conducted in a temperature‐controlled room maintained at 28°C ± 1°C.

### 2.3 NTT

The NTT is one of the main tests used to assess shy and bold behavior in zebrafish, allowing researchers to explore locomotor patterns and exploratory responses in a novel environment. In this test, one of the main parameters used to evaluate boldness is the time spent in the top of the tank, considering that fish tend to initially spend more time in the bottom and gradually explore the upper area, a zone with higher risk of predation in nature. Thus, bold fish tend to adapt faster, displaying increased exploration of the top, whereas shy fish tend to show a prominent geotaxis (Beigloo et al. 2024; Kalueff et al. 2013; Mustafa et al. 2019; Thörnqvist et al. 2019; Wong et al. 2010). Here, we used the NTT to assess baseline boldness levels for evaluating stress reactivity and working memory performance of bold and shy fish. Zebrafish boldness is a multifaceted trait influenced by multiple exploratory behaviors (Beigloo et al. 2024; Rajput et al. 2022) including distance from bottom and distance traveled. Thus, we calculated a boldness index considering two factors: distance from bottom (a constant parameter that reflects top exploration) and the distance traveled (an endpoint associated with overall activity). Increased distance from the bottom reflects boldness and a preference for the upper zone, which are hallmark traits of bold individuals, whereas distance traveled provides a measure of overall locomotion and exploratory drive. Integrating these metrics allowed us to account for both spatial and activity‐related aspects of boldness, providing a more comprehensive and accurate measure of this behavioral phenotype. Because parameters related to animal tank exploration have a higher weight than distance traveled, which is often assessed for locomotor analysis, the following formula was used: *Boldness index* = *0.67 × normalized distance from bottom + 0.33 × normalized distance*. Thus, both parameters were normalized, and distance from bottom had double the weight of distance traveled considering its relevance for distinguishing bold versus shy behavior. For the NTT, fish were individually placed in the experimental tank (25 *×* 5 cm; L *×* W with 10 cm of water depth) for 6 min (Egan et al. 2009; Parker et al. 2012; Rosemberg et al. 2012). A webcam was positioned horizontally at 37.5 cm from the tank (1920 *×* 1080 pixels, full HD), and the behavioral activity was analyzed using appropriate video‐tracking software (ANY‐maze, Stoelting Co., USA). Parameters such as distance traveled (m), maximum speed (m/s), absolute turn angle (°), and distance from bottom (m) were extracted. Immediately after the NTT, animals were exposed to the acute stressor.

### 2.4 Acute Stressor Exposure: CAS

CAS is a naturalistic fear cue released by zebrafish in response to predator attacks and serves to alert other fish of surrounding danger, effectively triggering stress‐related responses at physiological and behavioral levels (Abreu et al. 2016; Canzian et al. 2017; Fraker et al. 2009; Hall and Suboski 1995; Quadros et al. 2016; Speedie and Gerlai 2008; Wong et al. 2010). CAS was extracted by damaging epidermal cells with 15 shallow slices on both sides of a donor fish (euthanized previously by immersion in ice‐cold water) using a razor blade. After the epidermal cells were damaged, 10 mL of distilled water was mixed in a Petri dish covering the donor fish body (Canzian et al. 2017; Egan et al. 2009; Fontana et al. 2021b; Quadros et al. 2016; Speedie and Gerlai 2008). Animals were then exposed to water (CTRL group) or CAS in a dilution of 1.75 mL of the CAS extract in 500‐mL beakers for 5 min. To validate the effectiveness of the CAS preparation, each CAS extract was used for at least four fish, and all CAS‐exposed fish displayed consistent increases in stress‐related repetitive behaviors compared to controls. Immediately after CAS exposure, animals were tested on the FMP Y‐maze to evaluate their response to this acute stressor in terms of working memory and repetitive behaviors. A new batch of alarm substance was prepared for each experimental replicate (five replicates in total), with alarm substance from each donor fish used to expose up to six recipient fish at a time.

### 2.5 FMP Y‐Maze

The FMP Y‐maze task is a behavioral paradigm used to measure working memory and cognitive flexibility in zebrafish (Cleal et al. 2021; Fontana et al. 2019) and is also an important task for assessing repetitive behavior in this species (Fontana et al. 2021b). First, sequential turn data are converted into overlapping “tetragrams” (sequences of four choices). Tetragrams are very useful for FMP Y‐maze analysis because they capture sequences of four consecutive turns, allowing for detailed examination of repetitive versus alternating patterns in movement. This approach helps to identify predictable behavior patterns, which are indicative of decision‐making processes, critical in understanding cognitive function in the maze. Thus, data are separated in two analyses: (1) total percentage use (calculated as a proportion of total turns) of each of the two main “global” search strategies, alternations (LRLR + RLRL) and repetitions (RRRR + LLLL); and (2) “immediate” search strategy, analyzing the search pattern configurations over 10‐min time bins throughout 1 h of behavioral testing. Importantly, the global search strategy analysis is primarily used to assess working memory, as animals must recall their previous choices to navigate the maze. Alternations represent cognitively demanding choices, because they require the animal to remember its recent choices and inhibit the instinct to repeat familiar or recent behaviors. This process engages working memory and executive control, as the animal must actively track its movements to make a conscious, nonrepetitive choice, reflecting a higher level of cognitive processing. Alternation choices in the FMP Y‐maze are the most common strategy used across species, including zebrafish, mice, and humans (Cleal et al. 2021). Conversely, an increased use of pure repetitions (e.g., RRRR and LLLL) reflects reduced behavioral flexibility and has been associated with perseverative behaviors observed in animals exposed to acute stressor (Fontana et al. 2021b). Here, after the exposure to water or acute stressor, animals were individually tested in a glass Y‐maze tank covered with white adhesive paper. The Y‐maze tank consisted of three identical arms with no visual cues (5 cm length *×* 2 cm width; 120° angle between arms) filled with 10 cm of water depth. The maze did not contain any intra‐maze cues and ambient light was present to allow some visibility in the maze and a proper detection of the subject by automated video‐tracking software (~50 lx). FMP Y‐maze camera was positioned vertically at 37.5 cm from the water surface, and videos were recorded in a lower resolution considering the duration of the task (640 *×* 360 p). Animal behavior was analyzed based on their arm turns (left or right), and behavioral performance was assessed according to an overlapping series of four choices (tetragrams) for 60 min by extracting the sequence of arm choices from the video‐tracking software ANY‐maze (ANY‐maze, Stoelting Co., USA).

### 2.6 Statistics

Data normality and homogeneity of variance were analyzed by Kolmogorov–Smirnov and Bartlett's tests, respectively. The differences in the NTT for bold and shy fish were analyzed using a Student's *t*‐test. FMP Y‐maze total turns were analyzed using a two‐way ANOVA considering two factors: stress (two levels—CTRL *×* CAS) and baseline behavior (two levels—bold *×* shy). Frequency choice was similarly analyzed; however, it used tetragram choice (16 levels) as one of the main factors instead of stress. The rationale for using tetragrams in Y‐maze analysis lies in probability theory and sequence analysis. With four turns, there are 2^4^ = 16 possible tetragram sequences (e.g., RRRR, RRRL, RLRL, etc.). This finite set allows for a manageable but sufficiently diverse range of patterns to assess memory and decision processes. For analyzing the alternations and repetitions, a two‐way ANCOVA was performed, with total turns as a covariate, and stress and baseline behavior interactions were analyzed as main factors. Alternations and repetitions across time were analyzed using a repeated measures ANOVA with time bin (six levels—10–60 min) and group (4 levels—CTRL + Shy, CTRL + Bold, CAS + Shy, CAS + Bold) as main factors. Tukey's test was used for post hoc analysis, and results were considered significant when *p* ≤ 0.05. To investigate the relationships between the behavioral parameters and the individuals tested, a Pearson correlation analysis was conducted separately for two experimental groups: CTRL and CAS. The following behavioral parameters were selected for the correlation analysis based on their relevance to the tasks used here: distance traveled, maximum speed, absolute turn angle, distance from bottom, total turns, alternations, repetitions, and boldness index. Statistical analysis was performed using R Version 4.4.0 and visualized using *ggplot2*.

## 3 Results

### 3.1 Boldness Index Reflects Classical Behavioral Patterns Distinguishing Bold and Shy Animals

**FIGURE 2:**
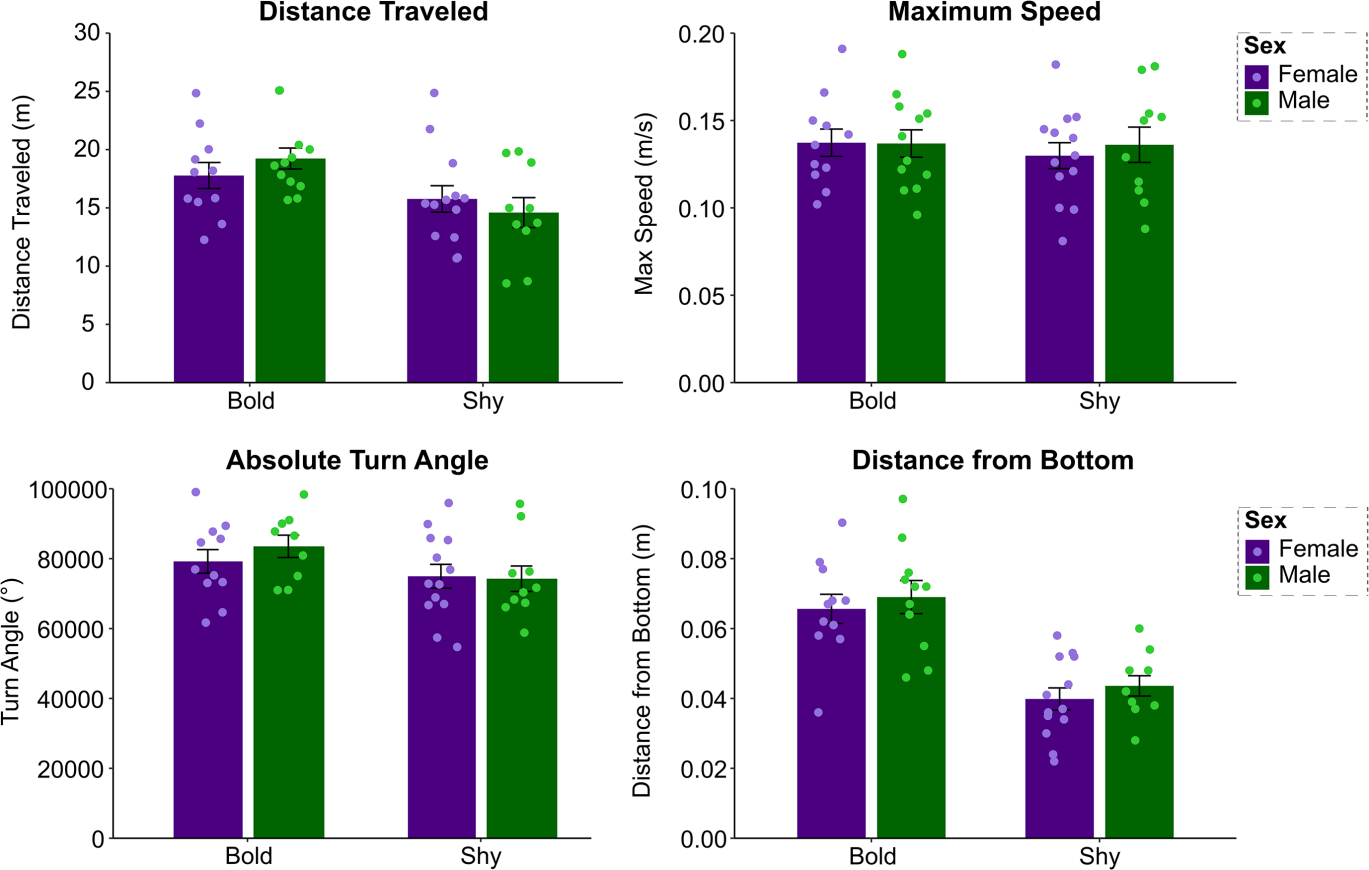
Behavioral responses of bold and shy fish separated by sex. No behavioral differences were found between sex for bold and shy fish (*p < 0.05; *n* = 11–13 per group). Data are represented as mean ± SEM. Differences between baseline behavior and sex were analyzed using a two‐way ANOVA, followed by Tukey's post hoc test for multiple comparisons.

Figure 1B shows the distribution of fish that were classified as bold or shy based on their boldness index. A balanced number of males and females were observed for both groups. Although males more often displayed higher boldness values, no significant differences were found between sexes (*p* = 0.067). No sex differences were also found in the overall analysis of distance traveled, maximum speed, absolute turn angle, and distance from bottom in the NTT following two‐way ANOVA comparing baseline behaviors and sex as main factors (Figure 2). Importantly, the sample size in this study may limit the detection of subtle sex differences, and caution should be taken in interpreting these results. To check the influence of classical NTT parameters on bold versus shy fish, we performed a direct comparison using Student's *t*‐test considering that those fish were not yet exposed to any other variable (i.e., stress exposure). Although no significant differences were found for maximum speed (*t*_44_ = 0.556, *p* = 0.58), distance traveled was significantly higher for bold fish compared to shy fish (*t*_44_ = 2.98, ***p = 0.005). Absolute turn angle (*t*_44_ = 2.68, **p = 0.01) and distance from bottom (*t*_44_ = 6.76, ****p < 0.001) were also significantly higher in bold fish compared to shy individuals, which reflects on fish exploratory activity of higher predation zones, an expected behavior considering how bold and shy fish were classified here (Figure 1C).

### 3.2 Bold Fish Show Different Patterns of Behavior on the FMP Y‐Maze Following Stress Exposure

**FIGURE 3:**
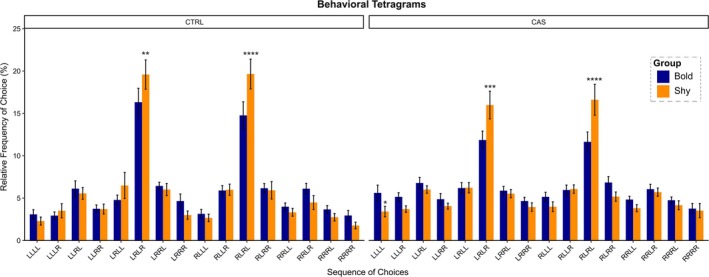
Behavioral tetragrams of control (CTRL) and conspecific alarm substance (CAS) groups, showing the differences between bold and shy fish. Data are represented as mean ± SEM and analyzed by two‐way ANOVA (frequency of choice * baseline behavior), followed by Tukey's post hoc test for multiple comparison. Asterisk represent significant difference compared to bold fish (*p < 0.05, **p < 0.01, ***p < 0.001, ****p < 0.0001; *n* = 11–12).

Based on fish baseline behavior (bold vs. shy), we first analyzed the behavioral tetragrams of choices after exposure to an acute stressor (Figure 3). Using two‐way ANOVA, we found a main effect for frequency of choice for both CTRL (*F*_1,320_ = 59.589, ***p < 0.001) and CAS (*F*_1,352_ = 37.101, ***p < 0.001) groups. Although no effects were found for baseline behavior (bold vs. shy), an interaction effect between frequency of choices and baseline behavior was found for CTRL (*F*_15,320_ = 2.098, **p = 0.009) and CAS (*F*_15,352_ = 3.437, ***p < 0.001). For the CTRL group, post hoc comparisons revealed that bold fish exhibited significantly lower LRLR (**p = 0.008) and RLRL values (***p = 0.0001) compared to shy fish, in which both frequency of choices is mainly correlated to working memory performance. A similar effect was observed for CAS, where lower values of LRLR (***p = 0.0001) and RLRL (****p < 0.0001) were observed for bold individuals when compared to shy fish. In response to stress, LLLL values were higher for bold fish compared to shy (*p = 0.035), a typical repetitive behavior pattern.

**FIGURE 4:**
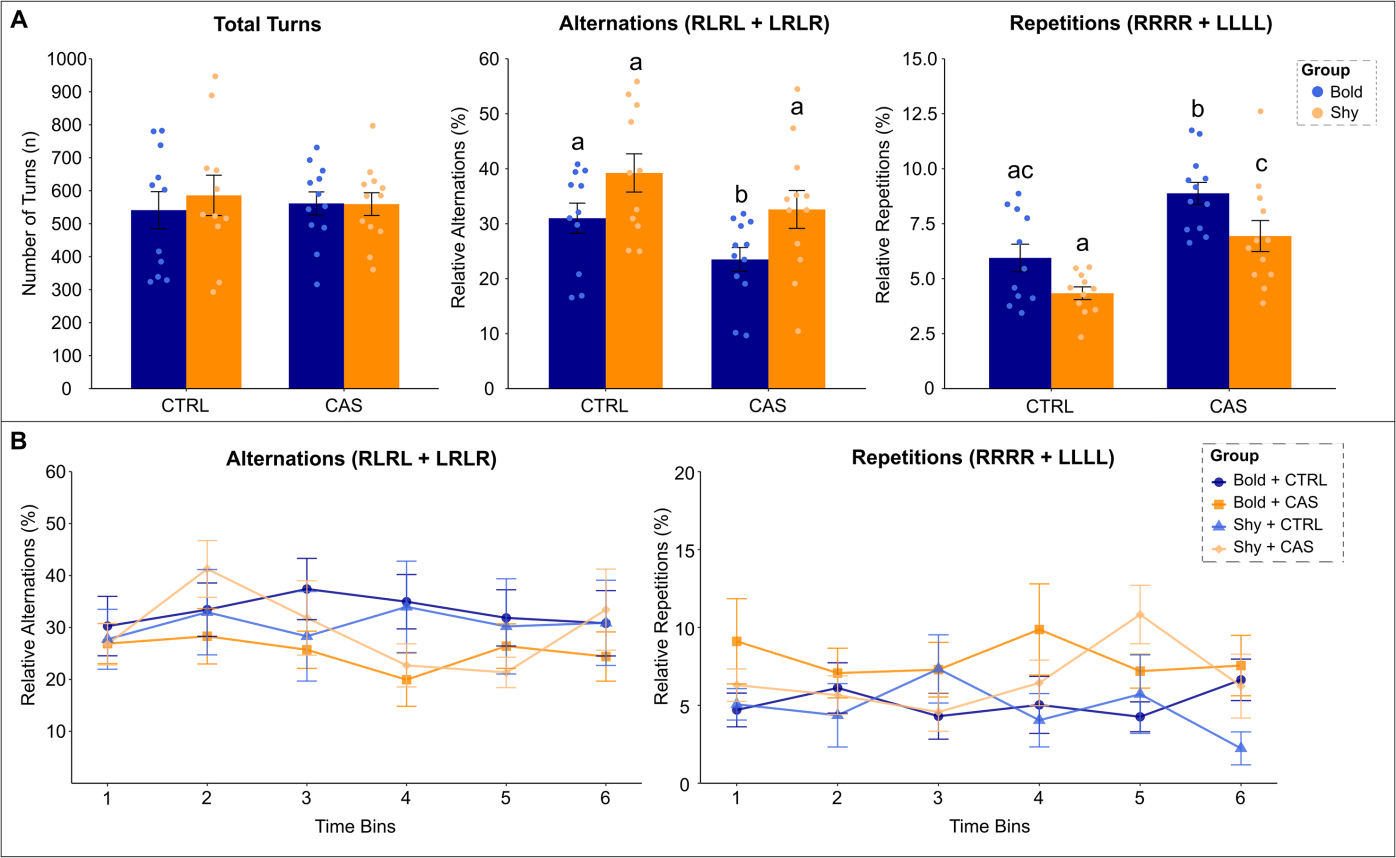
Main behaviors of bold and shy fish from control (CTRL) and conspecific alarm substance (CAS) groups in the FMP Y‐maze. (A) Overall analysis of the FMP Y‐maze for total turns, alternations, and repetitions displaying the interactions and differences between baseline behavior and stress responses. Data are represented as mean ± SEM and analyzed by two‐way ANCOVA (baseline behavior * stress exposure), followed by Tukey's post hoc test for multiple comparison. Different letters indicate significant differences among groups (*p < 0.05; *n* = 11–12). (B) Analysis across time for alternations and repetitions divided into six 10‐min bins. Data are represented as mean ± SEM and analyzed by two‐way RM‐ANOVA (group vs. time), followed by Tukey's post hoc test for multiple comparison (*n* = 11–12).

When further evaluating the effects of stress on bold and shy fish on the FMP Y‐maze, the analysis of total turns, alternations, and repetitions was performed (Figure 4). Total turns were not affected by both factors (baseline behavior and stress group). When two‐way ANCOVA was performed using total turns as a cofactor, there was a significant main effect of baseline behavior (*F*_1,41_ = 9.81, *p* = 0.003) and stress group (*F*_1,41_ = 6.48, *p* = 0.015), but no interaction between factors for alternations. Post hoc comparisons revealed that bold fish showed a significant decrease in alternations when comparing CTRL versus CAS groups (*p* = 0.048). Interestingly, shy fish showed higher alternation patterns compared to bold fish when looking at both CAS groups (*p* = 0.022). For repetitions, although no significant effects for interaction between baseline and stress group factors were found, a significant main effect of baseline behavior was observed (*F*_1,41_ = 9.98, *p* = 0.003), along with a significant main effect of stress group (*F*_1,41_ = 24.02, ****p < 0.0001). Tukey's post hoc test yielded a significant increase in repetitions when comparing CAS to CTRL bold (***p = 0.0007) and shy fish (****p < 0.0001). We also found a significant increase in repetitions for bold individuals when compared to shy animals (*p = 0.017) (Figure 4A).

A repeated measures ANOVA was conducted to examine the main effects of all groups considering baseline behavior and stress exposure across time (Figure 4B). No significant effects for time or group, or interaction between factors, were observed for alternations. Fish typically show a peak in their working memory around 40 min, and although we have not found this peak in CTRL animals, we found the lowest values of alternations at this point for CAS‐exposed fish, which could indicate a decline in animals' cognitive flexibility. Both bold and shy fish showed a similar pattern of behavior in terms of alternations across time. For repetitions, a main effect of group (*F*_3,261_ = 3.97, **p = 0.009) was found, indicating that the number of repetitions significantly varies between groups depending on the time point. Specifically, at 40 min (time bin 4), bold fish exposed to CAS showed a significant increase in repetitions compared to bold CTRL fish (*p = 0.048), in which a similar finding was observed for shy individuals (*p = 0.029). When looking at both CTRL groups, shy fish had a significant decrease in their repetitive behaviors compared to bold fish (*p = 0.015), a pattern that was not found for bold fish (which showed an increase in this pattern towards the last 10 min). Increased repetitions were found to be one of the main responses to stressors in the FMP Y‐maze, and interestingly, bold fish showed a peak in repetitions 10 min earlier than the shy fish, supporting a distinct pattern of behavior across time.

### 3.3 Higher Values of Boldness Index Are Associated With Decreased Alternations and Increased Repetitive Behaviors

**FIGURE 5:**
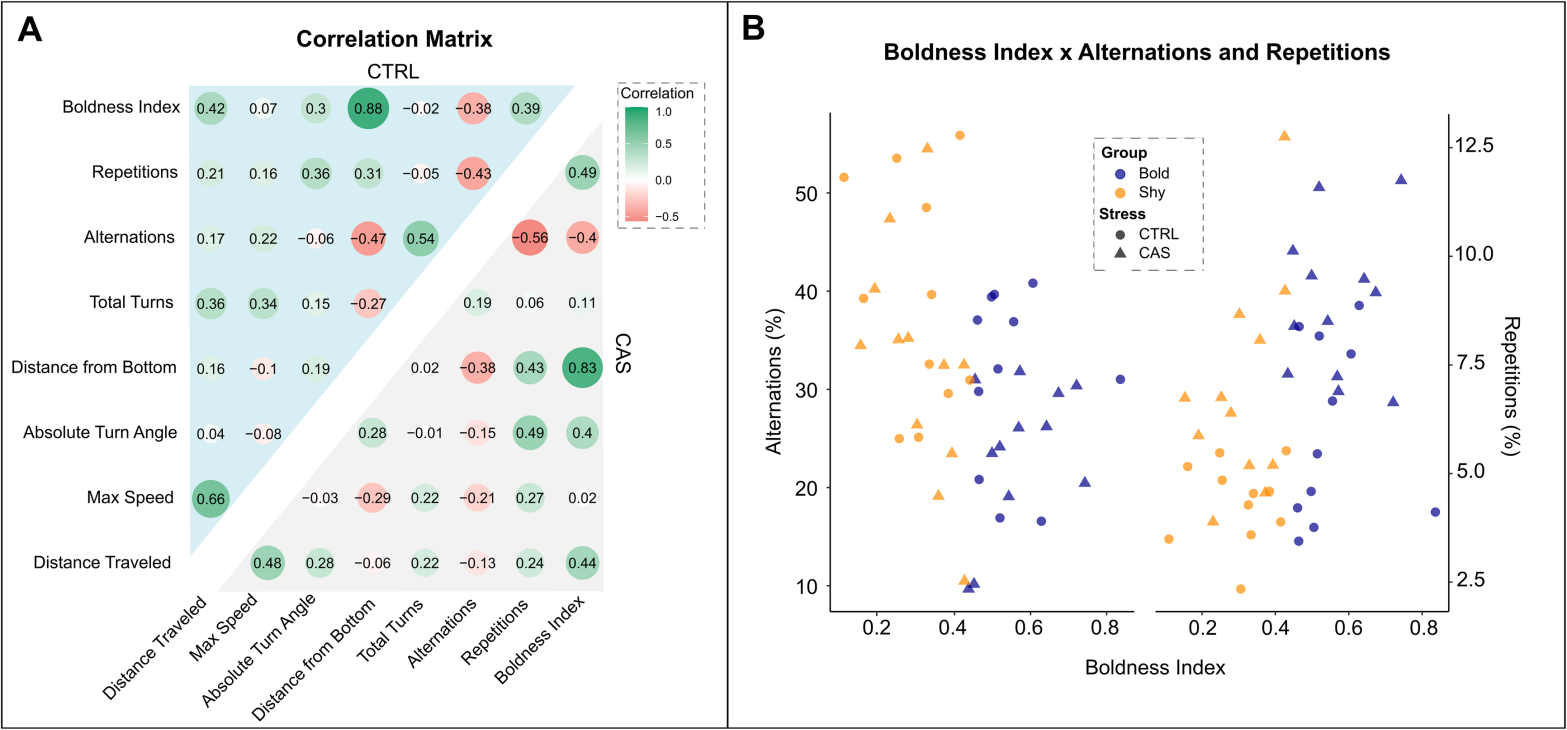
Association between boldness and main behaviors assessed in the experimental groups tested. (A) Correlation matrix for NTT and FMP Y‐maze parameters within CTRL (blue) and CAS (gray) groups, showing relationships between boldness index, total turns, alternations, repetitions, distance from bottom, and locomotor‐related parameters. Correlation coefficients are color‐coded from positive (green) to negative (red). (B) Scatter plot showing relationships between boldness index and percentages of alternations and repetitions, highlighting the distribution of different groups based on baseline behavior (bold vs. shy) and stress exposure (CTRL vs. CAS).

The putative association of boldness with the main behaviors measured was analyzed using Pearson's correlation test. Correlations with the absolute turn angle (*r* = 0.3 CTRL and 0.4 CAS) were found, corroborating previous information showing that bolder fish tend to not only swim more and explore riskier zones but also show higher turn angle values. When looking at the FMP Y‐maze parameters, this index was also negatively correlated with alternations (*r* = −0.38 CTRL and −0.40 CAS) and positively correlated to repetitions (*r* = 0.39 CTRL and 0.49 CAS), indicating that repetitive behaviors tend to increase as animals are bolder. An opposite effect was observed for working memory, in which animals with a higher boldness index have a reduced working memory. Repetitions were positively correlated to animals that had a higher absolute turn angle for both CTRL (*r* = 0.36) and CAS‐exposed fish (*r* = 0.49). Other important correlations can be observed for the first time here, in which stressed animals present a very low correlation of total turns with alternations (*r* = 0.19). However, in the CTRL group, total turns had a moderate correlation with animals' working memory performance when looking at alternations (*r* = 0.54) (Figure 5A). Figure 5B displays the interactions between the boldness index with alternations or repetitions by behavioral phenotype and stress group.

## 4 Discussion

Here, we provide new insights into how individual differences in boldness shape stress reactivity, repetitive behaviors, and working memory. By analyzing baseline behaviors in the NTT (bold vs. shy), we found that bold fish exhibit fewer alternations when looking at the frequency of choice, behavior correlated to working memory, regardless of acute stress exposure. This pattern was further supported by correlational analyses, showing that a higher boldness index was negatively correlated with alternations and positively correlated with repetitions. When exposed to CAS, both bold and shy individuals showed increased repetitive behaviors, confirming that stress induces repetitive behaviors in zebrafish from both groups. However, bold fish showed a more robust increase in repetitive behavior compared to shy fish after stress. Collectively, these data suggest that bold individuals may rely on simpler, habitual strategies. Repetitive behavior can indicate a lower cognitive load, as it requires less working memory and decision‐making effort than alternations, which demand active tracking of recent choices (Cleal et al. 2021). In bold individuals, who might naturally take more risks or act impulsively, the shift towards repetition under stress could reflect a coping mechanism, as repetitive behaviors are often linked to stress reduction in animals. This pattern may imply that boldness is associated with a more rigid response style when stressed, potentially limiting adaptability and cognitive flexibility in challenging environments.

Boldness and shyness are key behavioral traits that can shape how individuals react in challenging environments and in stressful situations (Sansom et al. 2009; Shaw 2020). The boldness index used in this study incorporates distance from the bottom and distance traveled, parameters that are well established in the literature for distinguishing bold and shy phenotypes (Beigloo et al. 2024; Rajput et al. 2022). These parameters are used considering that bold zebrafish are known to travel greater distances, reflecting increased overall exploration, as bolder animals are typically more active and prone to venturing into higher‐risk zones (e.g., upper zone) (dos Santos et al. 2023; Egan et al. 2009; Kalueff et al. 2013; Levin et al. 2007; Wong et al. 2010). Interestingly, we found that bold zebrafish exhibited increased absolute turn angle, likely reflecting more rapid changes in direction, which may indicate a higher willingness to take risks and investigate unfamiliar areas. Therefore, we highlight the boldness index as a reliable measurement for distinguishing bold and shy phenotypes, capturing the intricate complexity of boldness by considering multiple endpoints, such as proactive exploratory behavior and reduced caution in navigating the environment.

Boldness and shyness are also associated with other behavioral domains. For example, bold fish show higher aggression levels, being a consistent phenotype across two different behavioral contexts (Mamede et al. 2024). Bold fish also have a greater predisposition for taking risks in a new environments, presenting a faster habituation to novelty stress compared to shy fish (dos Santos et al. 2023). The behavioral difference in boldness also reflects on physiological changes, where proactive coping style (bold fish) was shown to have lower cortisol levels during the rising phase of the stress response compared with those from the proactive line (shy fish) (Wong et al. 2019). These findings indicate that boldness influences a range of behaviors beyond exploration, including aggressiveness, impulsiveness, and physiological responses to stress, with bold individuals generally prioritizing riskier, more proactive responses.

Impulsivity, often associated with boldness, can have a strong impact on cognitive tasks that require self‐regulation and decision‐making (Fortgang and Cannon 2022). For example, in humans, boldness negatively impacts executive functions, including working memory (Whitney et al. 2004). Working memory is an important cognitive process that involves the ability to retain and manipulate information over short periods of time (Cowan 2015). Similarly, we observed that bold fish showed a lower frequency of sequentially alternating choices compared to shy fish, independently of stress exposure. These behavioral patterns are more cognitively demanding, as they require continuous self‐monitoring and flexibility in switching between arm choices in the FMP Y‐maze (Cleal et al. 2021), which could indicate poor working memory in bold fish. Alternatively, this could also indicate a preference for more impulsive and less structured decision‐making strategies performed by bold fish, resulting in a less prominent alternated frequency of choices. In contrast, shy fish maintain these alternation patterns at higher levels, which could be due to their cautious choices and more deliberate decision‐making. Because shy fish are known to show reduced exploratory activity, their cautious exploration may allow them to monitor their environment closely, leading to use of alternations as a main escape response, which is a more cognitively demanding pattern. To corroborate these findings, correlations of the boldness index with alternations showed that the bolder phenotype is associated with the lowest alternation. Although considered a moderate correlation, this result highlights the potential cognitive impacts associated with impulsivity. However, boldness does not necessarily impact cognition negatively in tasks that may be more impulsively driven. For example, a previous study found a better performance of bold fish in conditioned place preference (CPP) and two‐choice tasks, showing a faster learning than shy fish (Corcoran et al. 2024), which could be a result of their lower neophobic tendencies and readiness to engage with novel stimuli. These tasks, which require rapid decision‐making rather than sustained self‐regulation, highlight how bold individuals' impulsivity may be advantageous in contexts that prioritize quick responses. Thus, boldness may reflect a trade‐off, where bold individuals, while potentially more explorative, have a lower cognitive efficiency in tasks that require sustained self‐regulation.

Working memory is also highly sensitive to stress, which can significantly impair the individual's ability to perform tasks that require sustained attention (Luethi et al. 2009). Although CAS did not change the overall alternations in shy fish, bold fish showed the worst cognitive performance in terms of working memory when acutely stressed. Our data not only show how stress can change working memory but also highlight the importance of studying individual differences, considering that only one behavioral phenotype was associated with this impairment. Although previous data showed that overall alternations are not affected by stress (Fontana et al. 2021b), the lack of changes in overall alternations could be due to the combined analysis of bold and shy animals within the same group, showing the influence of boldness on cognitively demanding tasks. Stressors also affect cognitive flexibility, where the lowest patterns of alternations are generally found at 40 min, the cognitive performance peak for zebrafish. Cognitive flexibility is crucial because it allows individuals to adapt to changing environments and tasks, making it essential for survival and problem‐solving (Cañas et al. 2003). Stress can severely impair cognitive flexibility, reducing the individual's capacity to learn from new information, therefore leading to the presence of other patterns as a strategy, such as repetitive behaviors (Fontana et al. 2021b).

Indeed, increased repetitions were found to be the main behavioral pattern shown in response to physical and chemical stressors (Fontana et al. 2021b). Repetitions are often seen as less cognitively demanding and tend to increase under stress conditions, indicating a shift towards more habitual responses (Smeets et al. 2023). Considering the individual differences of shy and bold in working memory and stress response, this raises important questions on how boldness affects the expression of repetitive behavioral sequences. Here, we found that both bold and shy groups respond to stress similarly by increasing repetitive patterns of behavior. These findings align with previous studies suggesting that repetitive behaviors, such as stereotypies, are often used as maladaptive coping mechanisms in response to stress (Langen et al. 2011; Ohtsubo et al. 2024; Zhang et al. 2022). However, when comparing the response of bold and shy fish to stress, bolder fish showed increased repetitions compared to shy fish after being exposed to an acute stressor. The differences found here may reflect a tendency of bold fish to display increased impulsive and less structured behaviors under stress, not only because of their lower alternation patterns but also considering their lower cognitive flexibility. Interestingly, although no differences were found for repetitions between bold and shy individuals, we observed a negative correlation of boldness index and repetitions, indicating that boldness levels can impact the fish's tendency to display repetitive behaviors. This association can be further explored considering the relationship of alternations and repetitions to boldness index, where bold animals tend to show higher repetitions like what is found only for shy fish when stressed. In humans and rodent models, boldness and impulsiveness have been previously linked with abnormal repetitive behaviors. For example, patients with psychiatric disorders, such as obsessive–compulsive disorder, also show higher impulsivity and sensation‐seeking, being often more prone to engage with repetitive and compulsive behaviors (Abramovitch and McKay 2016). In rats, dopamine transporter knockouts show alterations in decision‐making processes and in motivational states, as well as prominent motor stereotypies (Cinque et al. 2018). Zebrafish mutants for *adgrl3.1* gene, linked to ADHD, also show increased presence of repetitive behaviors in the FMP Y‐maze (Fontana et al. 2024) and boldness when approaching unfamiliar objects (Fontana et al. 2023). This gene was previously linked to alterations in the dopaminergic system in zebrafish (Lange et al. 2018, 2012), which emphasizes the role of this system in modulating key behavioral traits, such as boldness and motor stereotypes. Given the dopaminergic system's involvement in reward processing, motivation, and motor control, future studies examining its role in shy versus bold fish on the FMP Y‐maze can expand our understanding of how this system contributes to differences in cognitive flexibility, decision‐making, and exploratory behavior under stress.

Importantly, recent 3D analysis reveals that fish can exhibit up to four distinct behavioral phenotypes in response to novel environments, extending beyond just shy and bold responses, and that these phenotypes vary with strain and sex when larger sample sizes are used compared to conventional studies (Rajput et al. 2022). Therefore, the overall analysis of bold and shy fish in terms of working memory and repetitive behaviors in response to stress could be further explored to understand how additional behavioral phenotypes influence cognition and stress responses. Whereas this study focused on boldness and stress reactivity, future work with larger samples will be needed to explore how sex influences behavioral diversity and resilience. Although complex behaviors can be expanded beyond shy and bold, our work does shed a light on how the two main popular behavioral phenotypes used to understand zebrafish individual differences in novel environments influence working memory, repetitive behaviors, and response to stress.

## 5 Conclusions

Overall, our study demonstrates the critical role played by individual differences in shaping stress responses and cognitive outcomes. In particular, bold fish show a more disorganized pattern of behavior in the FMP Y‐maze by showing decreased alternated patterns through the analysis of behavioral tetragrams. Bold fish also showed an increased response to stress, in terms of the presence of repetitive behavioral sequences and a greater impact on their working memory. Shy fish still show similar patterns of behavior in response to stress by increasing repetitive behaviors, but no impact on working memory was observed. While exploring high‐risk zones and displaying pronounced locomotion, bold fish appeared less able to adapt to stress in cognitively demanding tasks, such as the FMP Y‐maze. This finding suggests that individual traits, such as boldness, can significantly modulate how animals cope with stress and how this affects other behavioral domains, such as memory and cognitive flexibility.

## Author Contributions

**Barbara D. Fontana:** conceptualization, data curation, formal analysis, funding acquisition, investigation, methodology, project administration, visualization, writing – original draft. **Hevelyn S. Moraes:** data curation, methodology, writing – review and editing. **Camilla W. Pretzel:** data curation, methodology, writing – review and editing. **Khadija A. Mohammed:** data curation, methodology, writing – review and editing. **Julia Canzian:** writing – review and editing. **Carla D. Bonan:** writing – review and editing. **Matthew O. Parker:** writing – review and editing. **Denis B. Rosemberg:** resources, supervision, writing – review and editing.

## Ethics Statement

All procedures were approved by the Institutional Animal Care and Use Committee (process number 7412110722).

## Conflicts of Interest

The authors declare no conflicts of interest.

## Peer Review

The peer review history for this article is available at https://www.webofscience.com/api/gateway/wos/peer‐review/10.1111/ejn.70173.

## Data Availability

Full data and the scripts used for data analysis are available on GitHub: github.com/BarbaraDFontana/Boldness_WorkingMemory_Zebrafish.
